# Addressing musculoskeletal curricular inadequacies within undergraduate medical education

**DOI:** 10.1186/s12909-024-05849-6

**Published:** 2024-08-06

**Authors:** Jason Peeler

**Affiliations:** 1 Department of Human Anatomy and Cell Science, Max Rady College of Medicine, Rady Faculty of Health Sciences, University of Manitoba, 745 Bannatyne Ave, Winnipeg, MB Canada; 2 Present Address: Pan Am Clinic Foundation, Winnipeg, MB Canada

**Keywords:** Pre-clerkship, Undergraduate medical education, Orthopaedics; physical medicine & rehabilitation, Rheumatology

## Abstract

**Background:**

Musculoskeletal (MSK) injuries and diseases place a significant burden on the health care system. Despite this, research indicates that physician training in the area of MSK medicine has historically been inadequate, with a majority of medical students feeling that their training in MSK medicine is lacking. The goal of this investigation was to evaluate the efficacy of a new preclinical MSK curriculum that was implemented within a nationally accredited allopathic medical program.

**Methods:**

Retrospective analysis was completed on five consecutive years (2017–2021) of preclinical MSK curricular data for 549 medical students, including mid and end-of-course examinations and end-of-course student satisfaction surveys. Both parametric and non-parametric methods of analysis were used to examine within and between class differences (*P* < 0.05).

**Results:**

The new MSK curriculum covered 15 of 16 “core or must know” topics in MSK medicine, and academic performance was consistently high over the 5-year period of analysis (final course marks ranged from 76.6 ± 7.1 to 81.4 ± 8.1; failures/year: range from 0 to 4), being equal or above levels of student performance observed for other courses delivered during preclinical studies. Likert data from end-of-course surveys demonstrated that feedback was overwhelmingly positive (overall course satisfaction ranged from a low of 3.07/4.00 to a high of 3.56/4.00) and indicated that students felt that the new preclinical MSK curriculum did effectively support medical student learning and knowledge retention.

**Conclusion:**

Results are expected to help advance the current body of knowledge that is dedicated to improving physician learning and knowledge retention in the area of MSK medicine and provides a curricular model that could be used by other nationally accredited medical programs to help enhance MSK learning at the preclinical levels of physician training.

**Supplementary Information:**

The online version contains supplementary material available at 10.1186/s12909-024-05849-6.

## Background

Musculoskeletal (MSK) injuries and diseases are among the most common medical conditions to be treated by a physician [1], accounting for more than 20% of emergency room visits [2], and approximately 30% of all primary care visits [3]. Data suggests that MSK conditions are among the leading causes of long-term disability [4]; have a profound impact on an individual’s quality of life, work productivity, social inclusion, and daily autonomy [5, 6]; and are becoming more prevalent as the life expectancy of our aging population increases [7]. Currently, the estimated annual cost associated with providing MSK care in the United States alone exceeds $900 billion [8].

Despite this significant burden of disease and large socioeconomic cost, previous research indicates that physicians lack adequate training in MSK medicine [9, 10], with many schools devoting only a very small fraction of their program’s total curricular time (< 3.0%) to topics related to MSK medicine [11–14]. Not surprisingly, research illustrates that a majority of medical students feel their training in MSK medicine is inadequate [15], and post-graduate data demonstrates that many physicians lack adequate confidence, knowledge, and clinical skills when practicing MSK medicine [16–18], with greater than 50% of practicing physicians failing to obtain a passing score when completing a standardized basic competency MSK examination after graduation [19–22].

While the preclinical education environment of medical schools in both Canada [23] and the United States [24] are rigorously evaluated, and initiatives designed to improve physician training in the area of MSK medicine have been introduced [25–29], recent evidence confirms that a high degree of variability and large inadequacies still exist in the preclinical MSK curricula of both AFMC (Association of Faculties of Medicine of Canada) and AAMC (Association of American Medical Colleges) accredited medical programs [13, 14]. The literature also indicates that there is a need for the identification and adoption of more consistent MSK content within the preclinical curricular of allopathic medical programs. Despite rigorous national accreditation standard for both Canadian and American medical programs, no gold standard currently exists for preclinical MSK curricula, and the efficacy of most, if not all, MSK curricula remains unknown.

**Fig. 1:**
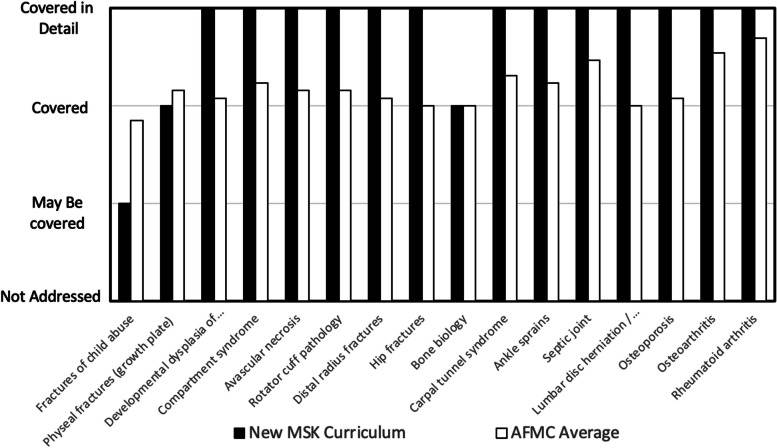
Core or must know topics in MSK medicine

To address inadequacies within its own preclinical MSK curriculum, a local AFMC accredited allopathic medical program had previously initiated a new preclinical MSK curriculum. The instructional time, organization, curricular content and modes of delivery implemented with this new course were adopted from published reports on previous MSK curricular initiatives, as well as expert-group recommendations for improving physician training in the area of MSK medicine [20, 25–31]. Interestingly, critical elements of this program’s new preclinical MSK curriculum were reflective of curricular averages recently published for both AFMC [14] and AAMC [13] accredited medical schools (Table 1). The program’s new MSK curriculum also “covered” or “covered in detail” fifteen of sixteen topics in MSK medicine that had been previously identified as “core or must know” content for physicians to know [32] (Fig. 1) – the one topic not adequately covered within the program's new preclinical curriculum was “fractures of child abuse”. Interestingly, when compared to other AFMC accredited medical programs, only the topics of “Physeal fractures” and “fractures of child abuse” were covered in more detail by the MSK curriculums of other programs [14].

**Table 1:** Preclinical curricular hours for nationally accredited allopathic medical programs

	**Current Program**	**AFMC Programs** [14]	**AAMC Programs** [13]
MSK Anatomy	30.0	29.8 (12 – 60)	29.7 (4 – 50)
MSK Medicine	68.0	58.0 (6- 204)	58.7 (6 – 150)
Clinical Skills	11.0	12.6 (3 – 40)	NA

Mean (Range)

As such, the purpose of this investigation was to retrospectively examine whether this nationally accredited medical program’s new MSK curriculum effectively addressed curricular inadequacies within the preclinical years of medical training. It was hypothesized that the new curriculum would support high levels of academic performance and course satisfaction among medical students. The results were expected to be highly generalizable to other AFMC and AAMC accredited medical programs, offer significant insight about how curricular inadequacies in MSK medicine could be effectively addressed at the preclinical level, as well as assist medical educators, program administrators, and accreditation organizations with establishing curricular standards for preclinical learning in MSK medicine. To quote Bernstein et al. (2011)…..“*now that musculoskeletal medicine is taught explicitly at most (US) schools, the enduring challenge is to ensure that it always is taught well*”(10).

## Methods

Following institutional ethics board approval (Ethics #: HS25704), de-identified MSK medicine course data for 549 medical students who had previously completed the new preclinical MSK curriculum were retrospectively analyzed from five consecutive years (2017 – 2021). Admission requirements for entry into this 4-year undergraduate allopathic medical program were the same for each year. All students completed the same thirty hour cadaveric—based MSK anatomy curriculum prior to beginning their preclinical studies in MSK medicine [33]. All students then completed the same stand-alone preclinical MSK medicine course during the second year of their training. The curriculum consisted of sixty-eight hours (h) of instruction that were organized and delivered as part of four course sections: 1). Upper extremity—16 h; 2). Lower extremity—15 h; 3). Spine—16 h; and 4). Rheumatology & chronic disease—15 h. The MSK curriculum also included an additional six hours of instruction on over-arching topics such as osteoarthritis (1 h), fracture management & healing (1 h), infectious disease (1 h), MSK development & genetics (2 h), and the burden of MSK disease (1 h). Finally, students were also required to complete eleven hours of “hands-on” instruction about MSK physical examination during their preclinical studies. These clinical skills sessions taught students to perform a basic patient history and MSK physical examination (5 h) and included practical learning sessions that introduced physical examination techniques that were specific to the upper extremity (2 h), lower extremity (2 h), and spine (2 h).

The same section leaders delivered the preclinical MSK curriculum over the entire 5-year period. The upper and lower extremity sections were each lead by a sports medicine physician (certified through the Canadian Association of Sport and Exercise Medicine); the spine section was directed by a physiatrist who was licensed through the Canadian Association of Physical Medicine & Rehabilitation (CAPM&R); and a Fellow of the Royal College of Physicians of Canada (FRCPC) qualified rheumatologist lead the rheumatology & chronic disease portion of the course. Each section leader possessed extensive teaching experience and had been in clinical practice for a minimum of 10 years. The same MSK medicine textbook (Essentials of Musculoskeletal Care—5th ed., Sarwark JF, American Academy of Orthopaedic Surgeons, 2010, ISBN: 978–0781175004-2), course notes package, and standards for academic advancement (a minimum final grade of 60%) were used over the 5-year period. Learning outcomes focused on “core or must know” topics in MSK medicine [13, 14, 32], providing detailed instruction about the clinical presentation, diagnosis, and non-surgical/surgical management of common MSK injuries and conditions. Beyond this, each year of the course also utilized the same modes of delivery for the sixty-eight hours of instruction: 1). Didactic lectures—37 h; 2). Small group case-based learning sessions—15 h; 3). Self-directed / asynchronous learning sessions—12 h; and 4). Formative testing sessions for each section of the curriculum—4 h.

Examinations were administered at the mid-point and on the final day of the preclinical MSK course using an ExamSoft online testing platform. Each exam consisted of a series of multiple-choice questions (each question was comprised of one stem statement and five distractor statements) that were mapped to session-specific learning objectives for each section of the curriculum. Distractor statements challenged student’s knowledge regarding a specific MSK injury/pathology and included content related to injury etiology and patient demographics, underlying patho-anatomy, clinical presentation, medical imaging, differential diagnosis, evidence-based methods of non-surgical/surgical management, and injury prognosis. The order of all multiple-choice questions on each individual student’s examination was randomized, and all multiple-choice questions were drawn from the same master bank of exam questions. The mid-course examination consisted of thirty-six multiple-choice questions, while the final examination included seventy-two multiple-choice questions and tested content that was delivered throughout the entire course. Embedded within examinations were a subset of identical multiple-choice questions that facilitated direct comparison of academic performance across consecutive years of the course.

Following the completion of the preclinical MSK course (but prior to final grades being released), students were asked to complete a course satisfaction survey that consisted of a standardized and validated set of fifteen questions [34] (the same end-of-course student survey was used with all preclinical courses) (See Appendix). Students were instructed to score each question using a simple 4-point Likert scale (Strongly Agree = 4; Agree = 3; Disagree = 2; Strongly Disagree = 1).

Data organization and statistical analysis were completed using Microsoft Excel (2019 edition) and VasserStats: Website for Statistical Computation (http://vassarstats.net). Repeated measures ANOVA testing (with Bonferroni post hoc analysis) was used to compare demographic data, and performance on mid-course and end-of-course examinations over the 5-year period. Direct comparisons of academic performance between years of the course were accomplished by analyzing an identical subset of multiple-choice questions that appeared on examinations over consecutive years of the course. Only questions with a difficulty index ≥ 0.30 and a discrimination index (point biserial) ≥ 0.20 were eligible to be selected for use on examinations over consecutive years. Non-parametric methods of analysis (Mann–Whitney U testing for the year to year comparison; Kruskal–Wallis testing for the multiple year comparison) were also used to compare the Likert-scale scoring from course evaluations for all five years of the course. Results were considered statistically significant if *P* < 0.05.

## Results

Table 2 summarizes the demographic data for each medical class over the 5-year period. In total, data from 549 medical students was analyzed. No significant differences were observed between cohort years when analyzing the adjusted grade point average (AGPA) or Medical College Admission Test (MCAT) score required for admittance into the medical program. Each year of medical class also demonstrated a similar division in gender (over the 5-year period, 270 males and 279 females were admitted), and the pattern of age distribution—with more than 50% of students in each medical class being between 18 and 23 years of age when starting medical school.

**Table 2:** Demographic Information arranged by incoming medical class

Demographic Data		Year 1	Year 2	Year 3	Year 4	Year 5
**Adjusted GPA Mean (range)**		NA	4.19 (3.54 – 4.50)	4.24 (3.72 – 4.50)	4.23 (3.72 – 4.50)	4.18 (3.34 – 4.50)
**MCAT score Mean (range)**		NA	513 (498 – 522)	514 (503 – 525)	514 (503 – 522)	514 (502 – 524)
**Gender**	**Female**	55	49	60	57	58
	**Male**	55	61	50	53	51
	**Another Gender Identity^**	n/a	n/a	n/a	0	1
**Age**	**18 – 23**	56	63	59	57	59
	**24—29**	46	41	45	48	44
	**30 + **	8	6	6	5	7

^*^ *P* < 0.05, ^ “Another Gender Identity” was added to gender selection criteria starting in 2019

Repeated measures ANOVA tests were used to compare the examination results for each year (Table 3.). Data indicated that there were no significant differences between medical classes when comparing mid-course or end-of-course examination results over the 5-year period. Despite a trend towards student performance on mid-course examinations being more variable than scoring on the end-of-course examination (as well as there being more mid-course examination failures), a between class comparison of mid-term and final exam results indicated that there were no significant differences in exam performance or the number of failures. Table 4 A, B, & C also provides further insight and a direct comparison of student performance on an identical subset of multiple-choice questions that were included on examinations over consecutive years of the course. ANOVA testing (with Bonferroni post hoc analysis) indicated that there were no significant differences in student performance on identical multiple-choice questions that were used over consecutive years. Having said this, results did suggest a trend towards student performance on rheumatology questions being consistently higher than performance on questions from other sections of the course. Finally, Table 5 compares final course marks (including number of failures) for all eleven Health and Disease courses that were delivered as part of the preclinical curriculum of this AFMC accredited medical program. While, a slight upward trend was observed in final MSK medicine course grades over the 5-year period, (year 1: 76.6% ± 7.1, 2 failures; year 2: 77.3% ± 8.9, 4 failures; year 3: 78.5% ± 9.0, 0 failures; year 4: 77.9% ± 9.9, 0 failures; and year 5: 81.4% ± 8.1, 1 failure), no statistically significant differences were observed in the final course grades (or number of course failures) over the 5-year period that was analyzed. Further, a year-by-year comparison of academic performance between all eleven courses delivered as part of this medical program’s preclinical curriculum also indicated that the MSK medicine course results were not significantly different from those for other preclinical courses taught in the same year.

**Table 3:** A comparison of exam performance (by percentage) in the preclinical MSK course

**Evaluation**		**Year 1** ***N* = 112**	**Year 2** ***N* = 108**	**Year 3** ***N* = 110**	**Year 4** ***N* = 107**	**Year 5** ***N* = 109**
**Mid-Course** **Exam** **36 MCQ**	**Class Average **(± Std Dev)	77.6 ±10.5	75.1 ±13.0	77.0 ±10.2	79.0 ±8.5	81.0 ± 10.1
	**Maximum**	97.2	97.2	97.0	97.2	96.9
	**Median**	77.7	77.7	79.4	80.6	81.3
	**Minimum**	52.7	27.7	47.0	58.3	40.6
	**# of fails**	9	12	9	3	3
**End-of-Course Exam** **72 MCQ**	**Class Average** (± Std Dev)	73.4 ±7.8	75.5 ±10.6	77.1 ±8.2	75.1 ±8.2	79.4± 9.5
	**Maximum**	90.6	94.0	94.3	95.7	97.2
	**Median**	74.5	77.5	77.4	75.7	76
	**Minimum**	46.6	0	57.7	55.7	40.9
	**# of fails**	5	5	4	5	4

**Table 4:** A,B,C. A comparison of student performance (by percentage) on identical MCQs over consecutive years of the preclinical MSK course

**Course Module**		**Year 1**	**Year 2**		**Year 3**	***P*** ** Value**
Upper Extremity *N* = 10		77.4 ± 15.4	79.7 ± 11.7		83.6 ± 11.9	0.57
Lower Extremity *N* = 11		73.5 ± 13.5	70.9 ± 17.3		69.7 ± 23.7	0.89
Spine *N* = 14		67.5 ± 23.5	76.0 ± 18.9		77.9 ± 18.3	0.36
Rheumatology *N* = 14		76.0 ± 13.6	81.9 ± 10.1		78.4 ± 18.1	0.56
All MCQs *N* = 49		73.3 ± 17.3	77.3 ± 15.1		77.4 ± 18.6	0.40
**Course Module**	**Year 2**		**Year 3**	**Year 4**		***P*** ** Value**
Upper Extremity *N* = 8	71.4 ± 20.7		80.1 ± 15.3	81.3 ± 16.2		0.48
Lower Extremity *N* = 6	76.2 ± 15.9		76.7 ± 26.9	77.3 ± 19.6		0.99
Spine *N* = 10	72.4 ± 21.2		73.2 ± 19.3	71.4 ± 22.4		0.98
Rheumatology *N* = 15	83.4 ± 10.2		81.0 ± 13.0	78.7 ± 15.0		0.61
All MCQs *N* = 39	77.0 ± 16.9		78.2 ± 17.3	77.1 ± 17.7		0.95
**Course Module**	**Year 3**		**Year 4**	**Year 5**		***P*** ** Value**
Upper Extremity *N* = 14	81.3 ± 12.2		82.0 ± 9.4	78.9 ± 12.8		0.76
Lower Extremity *N* = 10	78.2 ± 22.1		78.3 ± 18.1	77.9 ± 18.1		0.99
Spine *N* = 14	75.9 ± 13.2		72.7 ± 16.4	79.4 ± 12.7		0.46
Rheumatology *N* = 13	86.8 ± 7.6		85.9 ± 7.7	88.2 ± 7.1		0.73
All MCQs *N* = 51	80.6 ± 14.2		79.7 ± 13.9	81.2 ± 13.2		0.86

(mean ± standard deviation) There were no significant differences in academic performance between consecutive years of medical school on identical MCQs

^*^ *P* < 0.05

**Table 5:** A comparison of academic performance for all health & disease courses included in the preclinical curriculum

**Course Title** *- hours of instruction*	**Year 1**	**Year 2**	**Year 3**	**Year 4**	**Year 5**
Infectious Disease & Therapeutics *- 31 h*	83.3 ± 6.1 (0)	82.0 ± 7.0 (0)	85.1 ± 6.1 (0)	83.0 ± 7.3 (0)	83.1 ± 6.8 (1)
Cardiovascular *- 57 h*	77.6 ± 9.1 (4)	77.2 ± 10.1 (6)	77.4 ± 11.0 (6)	76.7 ± 10.7 (9)	80.2 ± 10.1 (6)
Respiratory *- 53 h*	75.8 ± 8.8 (4)	78.7 ± 9.8 (1)	78.7 ± 9.8 (3)	78.2 ± 8.1 (2)	80.1 ± 7.2 (0)
Oncology *- 13 h*	75.3 ± 10.7 (9)	86.5 ± 8.1 (1)	80.5 ± 9.6 (2)	77.4 ± 11.3 (4)	82.5 ± 8.9 (1)
Blood & Immunology *- 47 h*	72.7 ± 8.3 (5)	77.4 ± 7.4 (3)	76.0 ± 8.8 (5)	72.3 ± 9.3 (10)	76.4 ± 10.2 (4)
Neuroscience *- 108 h*	75.6 ± 6.6 (1)	75.9 ± 7.2 (2)	76.8 ± 9.6 (1)	77.1 ± 7.7 (2)	78.5 ± 8.7 (4)
♀ Reproductive Health *- 46 h*	82.0 ± 13.7 (0)	80.2 ± 7.0 (0)	82.2 ± 6.6 (0)	80.3 ± 7.4 (1)	81.6 ± 8.6 (1)
Endocrine & Metabolism *- 44 h*	80.3 ± 7.4 (0)	81.5 ± 7.8 (0)	81.7 ± 8.7 (0)	76.9 ± 9.0 (2)	81.7 ± 7.6 (1)
Gastroenterology, Hepatology & Nutrition *- 47 h*	80.0 ± 9.8 (1)	84.2 ± 7.7 (0)	82.6 ± 8.1 (0)	72.9 ± 11.7 (16)	79.4 ± 9.7 (5)
Urinary Tract *- 46 h*	83.4 ± 7.4 (0)	83.5 ± 9.0 (1)	83.3 ± 8.1 (2)	83.1 ± 7.6 (1)	83.4 ± 8.0 (0)
Musculoskeletal *- 68 h*	76.6 ± 7.1 (2)	77.3 ± 8.9 (4)	78.5 ± 9.0 (0)	77.9 ± 9.9 (0)	81.4 ± 8.1 (1)
***Yearly average (failures/year)***	***78.4% (26)***	***80.4% (18)***	***80.1% (19)***	77.8 (*47)	***80.8% (24)***

Final course mark – Mean percentage ± SD (including number of course failures)

^*^ *P* < 0.05

Likert scale data from end-of-course satisfaction surveys are presented in Table 6. Class response rates for the preclinical MSK course ranged from a low of 56% to a high of 84%. Student responses on each of the survey’s fifteen questions were extremely consistent over the 5-year period, with scoring dropping below 3.0 (ie. agree level) for only one question over the 5-year period – *“The course materials (notes, videos, handouts *etc*.) were well prepared and clearly explained”*. Despite there being a slight upward trend in student exam performance and final course marks over the 5-year period, non-parametric testing using both Mann–Whitney U (ie. year to year comparison) and Kruskal–Wallis (ie. multiple year comparison) testing indicated that there was no statistically significant difference (*P* < 0.05) in Likert scoring between medical classes on the survey questions. However, while “overall course satisfaction” scores for the 5-year period were consistently positive (ranging from a low of 3.07 to a high of 3.56), our analysis did indicate that the Likert scoring for years #4 & #5 of our analysis were significantly different than scores for the previous three years (*P* < 0.05).

**Table 6:** A comparison of course evaluation scores for the preclinical MSK course

Course Evaluation Questions	Year 1 *N* = 94	Year 2 *N* = 68	Year 3 *N* = 75	Year 4 *N* = 61	Year 5 *N* = 61	5-year mean
The objectives of the course were followed	3.16 ± 0.6	3.22 ± 0.6	3.28 ± 0.6	3.59 ± 0.6	3.58 ± 0.5	3.37
I learned and understood the subject matter of this course	3.15 ± 0.6	3.15 ± 0.6	3.04 ± 0.7	3.49 ± 0.5	3.54 ± 0.5	3.27
The use of lectures/WGS were effective in helping me learn	3.15 ± 0.6	3.16 ± 0.7	3.09 ± 0.8	3.61 ± 0.5	3.48 ± 0.7	3.30
The use of tutorials/ SGS were effective in helping me learn	3.28 ± 0.7	3.04 ± 0.8	3.45 ± 0.7	3.59 ± 0.6	3.62 ± 0.6	3.40
The use of other types of learning sessions (AS, sims, etc.) were effective in helping me learn	3.19 ± 0.6	3.15 ± 0.7	3.16 ± 0.7	3.58 ± 0.6	3.46 ± 0.6	3.31
The course encouraged me to integrate concepts from other courses	3.22 ± 0.6	3.18 ± 0.7	3.15 ± 0.7	3.52 ± 0.6	3.54 ± 0.6	3.32
Students in the class were encouraged to ask questions/participate	3.33 ± 0.6	3.30 ± 0.6	3.47 ± 0.5	3.70 ± 0.5	3.64 ± 0.5	3.49
There was appropriate assistance provided if needed	3.26 ± 0.6	3.27 ± 0.6	3.40 ± 0.5	3.69 ± 0.5	3.63 ± 0.5	3.45
There were opportunities to receive feedback about my progress during the course	3.22 ± 0.6	3.22 ± 0.7	3.35 ± 0.6	3.67 ± 0.5	3.58 ± 0.5	3.41
The content of this course was presented at a suitable pace	3.21 ± 0.6	3.15 ± 0.8	3.31 ± 0.6	3.56 ± 0.6	3.57 ± 0.5	3.36
The course materials (notes, videos, handouts etc.) were well prepared and clearly explained	3.06 ± 0.7	3.04 ± 0.7	2.96 ± 0.8	3.44 ± 0.6	3.42 ± 0.6	3.18
Lectures, whole group sessions, tutorials/labs and assigned studies were all well integrated	3.20 ± 0.7	3.24 ± 0.6	3.24 ± 0.8	3.62 ± 0.5	3.59 ± 0.5	3.38
The required or recommended readings were valuable	3.16 ± 0.7	3.19 ± 0.7	3.19 ± 0.8	3.51 ± 0.6	3.42 ± 0.6	3.29
Methods of assessing student work were fair and appropriate	3.06 ± 0.7	3.10 ± 0.7	3.16 ± 0.8	3.59 ± 0.5	3.49 ± 0.5	3.28
***Overall course satisfaction***	***3.09*** ** ± ** ***0.7***	***3.07*** ** ± ** ***0.5***	***3.08*** ** ± ** ***0.7***	*3.56 ** ± ** ***0.6***	*3.48 ** ± ** ***0.6***	***3.26***

A 4-point Likert scale (ranging from Strongly Agree = 4; Agree = 3; Disagree = 2; Strongly Disagree = 1) was used by students to rate their satisfaction with the course. (*SGS* small group session, *WGS* whole group session, *AS* assigned study, *sims* simulation lab); mean ± standard deviation; *P < 0.05

## Discussion

The results of this investigation provide important insight regarding the efficacy of a new preclinical MSK curriculum that was implemented at a local AFMC accredited allopathic medical program. Retrospective analysis of five consecutive years of course data involving a homogeneous group of medical students indicated that the new preclinical MSK curriculum supported consistently high levels of academic performance, with similar levels of within class variance observed over the 5-year period. A comparison of academic performance between the MSK course and other preclinical courses delivered over the same 5-year period also illustrated that MSK learning and knowledge retention among students was comparable to that observed for other preclinical courses (i.e., no significant differences when comparing final course grades or the number of course failures). Likert data from end-of-course evaluations also illustrated that student attitudes and perceptions about the learning environment created within the new MSK curriculum were consistently high over the 5-year period. As such, the results of this study offer valuable insight about how curricular inadequacies in MSK medicine may be successfully addressed at the preclinical level, and are expected to help advance the current body of knowledge that is dedicated to enhancing physician learning and knowledge retention in MSK medicine. The curriculum also serves as a model or template that could be followed by other AAMC and AFMC accredited medical programs who are engaging in curriculum reform, and hopefully will assist medical educators, program administrators, and accreditation bodies with the establishment of curricular standards for preclinical learning in MSK medicine.

Demographic data for this nationally accredited medical program indicated that the five medical classes that were analyzed for this investigation were very homogeneous, with the mean adjusted grade point average (AGPA) and Medical College Admission Test (MCAT) scoring being consistent across all years. Further, the distribution of each medical class by gender and age was highly uniform, and similar to data published for the seventeen Canadian medical schools in the annual AFMC report on Canadian Medical Education Statistics [35]. Because program entrance and accreditation requirements for both American (AAMC) and Canadian (AFMC) medical programs are globally similar (eg. AGPA, MCAT score, prerequisite degree, or specific coursework requirements), the data generated by this investigation are believed to be highly generalizable to the student population of other allopathic medical programs across North America.

In the present study, mid and end-of-course examination data was used to quantify MSK learning and knowledge retention associated with the new preclinical MSK curriculum. Data indicated that academic performance was consistently high over the 5-year period of analysis, with each year of medical students performing equally well on exam questions that were specific to each of the four sections of the MSK course, as well as on identical MCQs that were used on examinations over consecutive years. As such, according to the globally recognized Kirkpatrick Model of Training Evaluation (which rates training methods against four levels of criteria: Reaction, Learning, Behavior and Results) the new preclinical MSK curriculum successfully fulfilled the Reaction (which measures whether learners find the training engaging, favorable and relevant to their jobs) and Learning (which measures whether learners acquire the intended knowledge) levels of training [36]. This assertion is further reinforced by a comparison of academic performance (ie. final course marks, number of course failures) across all eleven Health and Disease courses delivered as part of this program’s preclinical curriculum which illustrated that academic performance during the MSK medicine curriculum was comparable to that of the other preclinical courses during the same time period. Finally, the trend towards exam performance on rheumatology questions being consistently higher than performance on questions from other sections of the curriculum is difficult to interpret. Anecdotally, past students have suggested that patterns of recognition for some inflammatory conditions may be easier for the new/novice MSK learner to identify (as compared to upper and lower extremity injuries such as a shoulder dislocation/separation or an anterior cruciate ligament injury of the knee), and the accurate diagnosis and management of many rheumatic conditions is often less reliant on a detailed understanding of MSK anatomy.

End-of-course Likert survey data illustrated a consistently high degree of overall course satisfaction among students (ranging from 3.07/4.00 to 3.56/4.00) over the 5-year period. Scoring for all fifteen survey questions only dipped below the “agree” level (or 3.00/4.00) once during this time, and scoring averages for each of the fifteen questions were extremely consistent over the 5-year period (ranging from a low of 3.18/4.00 for the survey question “*The course materials (notes, videos, handouts, *etc.*) were well prepared and clearly explained*” to a high of 3.49/4.00 for the survey question “*Students in the class were encouraged to ask questions/participate*”). The within and between year homogeneity in survey scoring suggested that students felt very positive about the preclinical MSK learning environment, believing that the course organization, number of contact hours, interdisciplinary and multimodal methods of delivery effectively supported their ability to learn and retain knowledge about “core or most know” topics in MSK medicine. Interestingly, the overall level of course satisfaction among students did significantly increase in last two years of our analysis. This finding is difficult to interpret, as no significant changes were made to the MSK curricular content or organization over the 5-year period of analysis. However, the MSK course is scheduled as the last preclinical course prior to students entering clerkship. As such, student fatigue and anxiety are always extremely high. Because of this, the preclinical MSK course director had more recently made a concerted effort to enhance communication and interactions with the student body, being more receptive and empathetic to student concerns as they arise on a day-to-day basis during the delivery of the MSK curriculum (rather than after completion of the course).

Class response rates for the end-of-course survey were also high, with approximately 2/3rds (66%) of the 549 students who completed the new preclinical MSK curriculum responding over the 5-year period of analysis. Previous research suggests that medical student survey response rates can be highly variable [37, 38], and dependent on a host of factors including (but not limited to) the length of the questionnaire, timing within the academic schedule, and the year of study within the medical program. The willingness of students at this nationally accredited medical program to provide course feedback is likely the result of three main factors: 1). Ease of survey completion: End-of-course surveys were administered electronically through an online student portal which facilitated quick and easy survey completion as part of a student’s regular post-course routine while awaiting release of their final course mark; 2). Student feedback is valued by this AFMC accredited medical program: Students at this institution are aware of the important role that their feedback plays in AFMC accreditation process; and 3). Survey results are included in ongoing course review: End-of-course surveys are an essential component of this medical program’s annual course review process during which course survey data are reviewed and discussed by both senior administrators, preclinical course directors, and representatives of the student body.

To the author’s knowledge, this investigation is among the first to retrospectively evaluate the efficacy of a new preclinical MSK curriculum over an extended period. The total instructional hours, content, and organization of the new curriculum were based on the key recommendations made by experts in the field of MSK medicine [31, 32, 39–41], and the curriculum was delivered in a manner that was reflective of many of the current pedagogical trends that are being observed within revised medical curriculums from around the world [13, 41, 42]. While a host of factors can influence the successful implementation of a new curriculum, the author believes that there were several critical factors which contributed to the efficacy of this new preclinical MSK medicine course. These include:

1. *Students developed a strong foundation in MSK anatomy prior to enrollment in the MSK medicine curriculum* – In the current program, all students completed a thirty-hour cadaveric-based MSK anatomy course prior to beginning the preclinical MSK medicine curriculum in the 2nd year of medical studies. The anatomy course supported student learning using radiological correlates, clinical cases, and functional anatomy tutorials, which served to reinforce “core or must know” MSK anatomy knowledge vital for future clinical learning in MSK medicine. This anatomy curriculum has been previously reported to support high levels of academic performance and student engagement among preclinical students [33, 43]. Additionally, previous research confirmed that the total instructional time allocated for this program’s MSK anatomy course was higher than the average times recently published for accredited medical programs in United States [13] and Canada [14].
2. *Adequate MSK instructional time was provided within the preclinical curriculum –* Previous research clearly indicates that medical programs have historically allocated only a very small percentage of curricular time (< 3%) to instruction on topics related to MSK medicine [9, 10]. Within the current program, the new MSK curriculum represented approximately 12% of the total curricular hours that were allotted to Health and Disease education within the preclinical curriculum. In fact, neuroscience was the only Health and Disease course to provide students with more instructional time during preclinical education. Additionally, the MSK curricular time (68 h) was far superior to course hours previously reported by other medical programs [11, 12], and closely aligned with the curricular averages recently reported for accredited medical programs within the both the United States (mean = 58.7 h, range 6 – 150) [13] and Canada (mean 58.0 h, range 6 – 204) [14].
3. *The preclinical curriculum focused on “core or must know” topics in MSK medicine* – Research indicates that there is a glaring lack of consistency in the MSK topics that are included in the preclinical MSK curriculums of medical schools [10, 12, 19, 31, 37, 44], with preclinical MSK curricular data illustrating that less than 25% of “core or must know” MSK topics are reliably covered by accredited medical programs in North America [13, 14]. In contrast, the new preclinical MSK curriculum of the current program “covered” or “covered in detail” 15 out of 16 (94%) MSK topics that research and expert opinion have previously identified as critical topics in MSK medicine—the only topic not “covered” or “covered in detail” was “fractures of child abuse” [13, 21, 32, 45, 46].
4. *MSK learning was supported by consistent and complimentary learning opportunities using an interdisciplinary and multi-modal approach to curriculum delivery* -  

This new preclinical MSK curriculum was delivered in a very consistent and organized manner over the 5-year period. The Course Director and four section leaders for the curriculum were highly knowledgeable and experienced clinical educators, who helped to create a consistent and predictable learning environment that had great continuity. They worked together to provide learners with an ordered sequence of complimentary learning opportunities which supported the delivery of a standardized set of learning outcomes that focused on “core or must know” topics in MSK medicine. These learning outcomes were each linked or mapped to a series of MCQs that were contained in a preclinical exam bank. All MSK exam questions had been previously validated by the MSK section leaders, and psychometric data was available for all MSK exam questions. The preclinical MSK curriculum utilized a multi-modal and interdisciplinary method of delivery that was supported by a standardized course notes package that included evidence-based supplemental readings, and answer keys for all self-guided learning, case studies, and formative evaluation sessions. All didactic lectures were delivered by one of the four MSK section leaders (this ensured continuity), while small group learning sessions were led by a variety of different “physician types” who work in the field of MSK medicine (i.e., orthopaedics, rheumatology, physical medicine & rehabilitation, radiology, sports medicine and family medicine). An interdisciplinary approach to preclinical MSK learning has been a recommendation of previous investigations about MSK medicine curriculums [13, 31, 47, 48], and the author believes that the high levels of academic performance and overall course satisfaction that were observed for this new preclinical MSK curriculum were at least in part attributable to the fact that the course was taught by an interdisciplinary group of physicians who provided students with a board perspective and greater understanding of how differences in physician training and areas of practice can influence a clinician’s approach to the assessment and management of “core or must know” MSK injuries / pathologies..

This study is not without limitation. Program restrictions related to academic progression and student privacy prevented investigators from directly comparing an individual student’s academic performance in the MSK medicine course with that of the other courses delivered during the preclinical years of training. This included the analysis of data from OSCE-type examinations which are used to evaluate MSK clinical skills throughout a student’s four years of medical training. It is also possible that academic performance in other areas of the preclinical curriculum may have influenced student confidence and academic performance during the MSK medicine course. Beyond this, it should be acknowledged that data from end-of-course student surveys may have been influenced by a response bias – with students who enjoyed the MSK topic and excelled at comprehending the course materials being more likely to complete the end-of-course survey and rate the course positively. Also, the use of “institution specific” data from mid and end-of-course examinations, as well as end-of-course student evaluations, likely precludes comparison of this data with that from other MSK investigations. Finally, this retrospective analysis was limited to the preclinical years of MSK learning, and it is beyond the scope of this investigation to draw conclusions about how academic performance during the preclinical years of study may influence MSK learning and knowledge retention that occurs during the clerkship or the post-graduate years of physician training. Having said this, data from this investigation can be used to support future investigations designed to explore long term MSK knowledge, confidence and clinical competencies among post-graduate physicians from this program, as well as other AFMC and AAMC accredited programs.

## Conclusions

Research clearly indicates that physician’s lack adequate knowledge, confidence, and clinical skills when practicing MSK medicine [25, 31, 40]. Despite ongoing initiatives designed to improve physician training in the area of MSK medicine [25–29], recent evidence confirms that a high degree of variability and large inadequacies still exist in the preclinical MSK curricula of both AFMC [14] and AAMC [13] accredited medical programs. To address inadequacies within its own preclinical MSK curriculum, a local nationally accredited allopathic medical program had previously initiated a new preclinical MSK curriculum. The goal of the current investigation was to retrospectively investigate the efficacy of this new preclinical MSK curriculum. Results illustrated that the new course supported consistently high levels of academic performance for a homogeneous group of preclinical medical students, and that student’s perceptions and attitudes towards the new curriculum were overwhelmingly positive. Additionally, when data was compared against other Health and Disease courses delivered within the program’s preclinical years, student learning and knowledge retention for the MSK curriculum was equal or superior to that observed for other courses. These results are expected to help advance the current body of knowledge that is dedicated to improving physician learning and knowledge retention in the area of MSK medicine and provide a curricular model that could be used by other AAMC and AFMC accredited medical programs to help enhance MSK learning and knowledge retention during the preclinical levels of physician training. Finally, it is hoped that the data from this longitudinal investigation will assist medical educators, program administrators, and accreditation bodies with the establishment of more consistent curricular standards for MSK medicine in the preclinical years of physician training.

### Supplementary Information

Supplementary Material 1.

## Data Availability

The datasets used and/or analysed during the current study are available from the corresponding author on reasonable request.

## Abbreviations

AAMC: Association of American Medical Colleges
AFMC: Association of Faculties of Medicine of Canada
AGPA: Adjusted grade point average
AS: Assigned study
CAPM&R: Canadian Association of Physical Medicine & Rehabilitation
FRCPC: Fellow of the Royal College of Physicians of Canada
MCAT: Medical college admissions test
MCQs: Multiple choice questions
MSK: Musculoskeletal
SIMS: Simulation lab
SGS: Small group session
WGS: Whole group session
