# Supplementary material for: Addressing musculoskeletal curricular inadequacies within undergraduate medical education
Source: BMC Med Educ. 2024 Aug 6;24:845. doi: 10.1186/s12909-024-05849-6 (PMC11304564; doi:10.1186/s12909-024-05849-6)
Supplement: Supplementary file 1 — Supplementary Material 1. [file 12909_2024_5849_MOESM1_ESM.docx]

Appendix – Course Satisfaction Survey

| **Course Evaluation Questions** | | **Strongly Disagree** | **Disagree** | **Agree** | **Strongly Agree** |
| --- | --- | --- | --- | --- | --- |
| The objectives of the course were followed. | | 1 | 2 | 3 | 4 |
| I learned and understood the subject matter of this course. | | 1 | 2 | 3 | 4 |
| The use of lectures/WGS were effective in helping me learn. | | 1 | 2 | 3 | 4 |
| The use of tutorials/ SGS were effective in helping me learn. | | 1 | 2 | 3 | 4 |
| The use of other types of learning sessions (AS, sims, etc.) were effective in helping me learn. | | 1 | 2 | 3 | 4 |
| The course encouraged me to integrate concepts from other courses. | | 1 | 2 | 3 | 4 |
| Students in the class were encouraged to ask questions / participate. | | 1 | 2 | 3 | 4 |
| There was appropriate assistance provided if needed. | | 1 | 2 | 3 | 4 |
| There were opportunities to receive feedback about my progress during the course. | | 1 | 2 | 3 | 4 |
| The content of this course was presented at a suitable pace. | | 1 | 2 | 3 | 4 |
| The course materials (notes, videos, handouts etc.) were well prepared and clearly explained. | | 1 | 2 | 3 | 4 |
| Lectures, whole group sessions, tutorials/labs and assigned studies were all well integrated. | | 1 | 2 | 3 | 4 |
| The required or recommended readings were valuable. | | 1 | 2 | 3 | 4 |
| Methods of assessing student work were fair and appropriate. | | 1 | 2 | 3 | 4 |
| ***My overall assessment of this course is:*** | 1  Unsatisfactory | 2  Satisfactory | 3  Good | 4  Very Good | 5  Excellent |

A 4-point Likert scale (ranging from Strongly Agree = 4; Agree = 3; Disagree = 2; Strongly Disagree = 1)
